# Supplementary material for: DeepAllo: allosteric site prediction using protein language model (pLM) with multitask learning
Source: Bioinformatics. 2025 May 15;41(6):btaf294. doi: 10.1093/bioinformatics/btaf294 (PMC12145174; doi:10.1093/bioinformatics/btaf294)
Supplement: btaf294_Supplementary_Data [file btaf294_supplementary_data.pdf]

DeepAllo: Supplementary Material

Moaaz Khokhar, Ozlem Keskin, Attila Gursoy

S1. Proteins Dataset with Names and PDB IDs

Table S1: List of 207 proteins and their PDB IDs in alphabetical order.

No.	Protein Name	PDB ID
1	Protein (Bovine Seminal Ribonuclease)	11BG
2	Carbamoyl Phosphate Synthetase (Large Chain)	1A9X
3	Glutamine Phosphoribosylpyrophosphate Amidotransferase	1AO0
4	Alpha-Amylase	1AQM
5	Protein (5-Aminolevulinic Acid Dehydratase)	1B4K
6	Protein (Purine Repressor)	1BDH
7	Tryptophan Synthase	1BEU
8	Serine Hydroxymethyltransferase, Cytosolic	1BJ4
9	Phosphoribosyl Pyrophosphate Synthetase	1DKR
10	Atrial Natriuretic Peptide Receptor A	1DP4
11	Lac Repressor	1EFA
12	Cytochrome P450Eryf	1EUP
13	Rad50 Abc-Atpase	1F2U
14	O-Acetylserine Sulfhydrylase	1FCJ
15	Phosphoenolpyruvate Carboxylase	1FIY
16	Receptor-Type Adenylate Cyclase Gresag 4.1	1FX2
17	Hemoglobin Alpha Chain	1G9V
18	Annexin V	1HAK
19	Anaerobic Ribonucleotide-Triphosphate Reductase	1HK8
20	S-Adenosylmethionine Decarboxylase Beta Chain	1I72
21	Anthranilate Synthase	1I7S
22	3-Deoxy-D-Arabino-Heptulosonate-7-Phosphatesynthase	1KFL
23	L-Lactate Dehydrogenase	1LDN
24	Tryptophan-Trna Ligase	1M83
25	Sulfate Adenylyltransferase	1M8P
26	Cyclic Nucleotide Phosphodiesterase 2A	1MC0
27	S-Adenosylmethionine Decarboxylase Proenzyme	1MSV
28	Fk506-Binding Protein	1NSG
29	Amine Oxidase [Flavin-Containing] B	1OJ9
30	Ribonucleoside-Diphosphate Reductase 2 Alphachain	1PEQ
31	Nad-Dependent Malic Enzyme, Mitochondrial	1PJ2
32	Protein (Hexokinase)	1QHA
33	Parathion Hydrolase	1QW7
34	Repressor Protein	1R1V
35	Integrin Alpha-L	1RD4
36	Caspase-7	1SHL
37	Protein-Tyrosine Phosphatase, Non-Receptor Type 1	1T48
38	Cgmp-Specific 3',5'-Cyclic Phosphodiesterase	1TBF
39	Atp-Dependent Clp Protease Atp-Binding Subunit Clpx	1UM8
40	B-Raf Proto-Oncogene Serine/Threonine-Protein Kinase	1UWH
41	Glyceraldehyde-3-Phosphate Dehydrogenase (Nadp+)	1UXN
42	Beta-Lactamase Shv-1	1VM1
43	Uracil Phosphoribosyltransferase	1VST
44	Cytochrome P450 3A4	1W0F
45	Acetyl-Coenzyme A Carboxylase	1W96
46	Hut Operon Positive Regulatory Protein	1WPU
47	Ribonucleotide Reductase, B12-Dependent	1XJE
48	Reca Protein	1XMV

Continued on next page

Table S1 – continued from previous page

No.	Protein Name	PDB ID
49	Arginine Repressor	1XXB
50	Coagulation Factor VII	1YGC
51	Allantoate Amidohydrolase	1Z2L
52	Copper-Containing Nitrite Reductase	1ZDS
53	Dna-Directed Rna Polymerase Alpha Chain	2A69
54	Divalent Cation Transport-Related Protein	2BBH
55	Acetylglutamate Kinase	2BTY
56	Pyruvate Dehydrogensae Kinase Isoenzyme 2	2BU2
57	Aspartokinase	2CDQ
58	Protein (Arginase)	2CEV
59	Protein-Arginine Deiminase Type Iv	2DEW
60	235Aa Long Hypothetical Biotin-[Acetyl-Coa-Carboxylase]Ligase	2DJZ
61	Bira Bifunctional Protein	2EWN
62	Dna Repair And Recombination Protein Rada	2FPL
63	Estrogen Receptor Beta	2FSZ
64	Deoxycytidylate Deaminase	2HVW
65	D-Alanine-D-Alanine Ligase	2I80
66	Acetylcholinesterase	2J3Q
67	Glutamate Racemase	2JFN
68	Glutamate Racemase	2JFZ
69	Myosin-2 Heavy Chain	2JHR
70	DNA Mismatch Repair Protein MSH2	2O8D
71	Thymidylate Synthetase	2ONB
72	Nitric Oxide Synthase, Inducible	2ORO
73	Virulence Factor Regulator	2OZ6
74	L-Asparaginase I	2P2N
75	Arginine Repressor	2P5M
76	D-3-Phosphoglycerate Dehydrogenase	2PA3
77	Kinesin-Like Protein Kif11	2PG2
78	Isomerase Domain Of Glutamine-Fructose-6-Phosphatetransaminase (Isomerizing)	2POC
79	Hyperpolarization-Activated (Ih) Channel	2PTM
80	Phenylpyruvate Decarboxylase	2Q5Q
81	Transporter	2Q6H
82	Fructose-Bisphosphatase	2Q8M
83	Caspase-7	2QLJ
84	Prephenate Dehydratase	2QMX
85	Uncharacterized Protein Vca0042	2RDE
86	Amp-Activated Protein Kinase Catalytic Subunit Alpha-1	2V8Q
87	Atp Phosphoribosyltransferase	2VD3
88	Bifunctional Protein Glmu	2VD4
89	Pyruvate Decarboxylase Isozyme 1	2VK1
90	Tetracycline Resistance Repressor Protein	2VPR
91	Ribonucleoside-Diphosphate Reductase Largesubunit	2WGH
92	Glucosamine-6-Phosphate Deaminase	2WU1
93	2-Oxoglutarate Decarboxylase	2Y0P
94	Nickel And Cobalt Resistance Protein Cnrr	2Y39
95	2-C-Methyl-D-Erythritol 4-Phosphate Cytidylyltransferase,Chloroplastic	2YC3
96	Androgen Receptor	2YLO
97	Glycogen Phosphorylase, Liver Form	2ZB2
98	Putative Uncharacterized Protein Ph0207	3ATH
99	Glucose-1-Phosphate Adenylyltransferase	3BRK
100	Glutamate Receptor, Ionotropic Kainate 1	3C35
101	Protein RECA	3CMW
102	Transthyretin	3CN0
103	Heat Shock Protein Homolog Sse1	3D2E
104	Putative Acetylglutamate Synthase	3D2P
105	Cone Cgmp-Specific 3',5'-Cyclic Phosphodiesterase Subunitalpha'	3DBA
106	Carbonic Anhydrase 2	3E2A
107	Cyclic Nucleotide-Binding Protein	3E5U

Continued on next page

Table S1 – continued from previous page

No.	Protein Name	PDB ID
108	Dual Specificity Mitogen-Activated Protein Kinase Kinase 1	3EQC
109	Glud1 Protein	3ETD
110	Endothelial Pas Domain-Containing Protein 1	3F1O
111	Alpha-Isopropylmalate Synthase	3F6H
112	Leukotriene A-4 Hydrolase	3FUD
113	Rtx Toxin Rtxa	3GCD
114	Rna-Directed Rna Polymerase	3GNV
115	Casein Kinase Ii Subunit Alpha	3H30
116	Exodeoxyribonuclease I	3HL8
117	Protein Fimx	3HV8
118	Reverse Transcriptase/Ribonuclease H	3I0S
119	Transcriptional Regulator, Crp/Fnr Family	3I54
120	Camp-Specific 3',5'-Cyclic Phosphodiesterase 4D	3IAD
121	Macrophage Migration Inhibitory Factor	3IJG
122	Serine/Threonine-Protein Kinase Chk1	3JVS
123	Peroxisome Proliferator-Activated Receptor Gamma	3K8S
124	Protease	3KF0
125	Probable 3-Deoxy-D-Arabino-Heptulosonate 7-Phosphatesynthase Arog	3KGF
126	Protein Mj1225	3KH5
127	Caspase-3	3KJF
128	Aspartokinase	3L76
129	Global Nitrogen Regulator	3LA3
130	Arginine Repressor	3LAP
131	Isocitrate Dehydrogenase Kinase/Phosphatase	3LCB
132	Serum Albumin	3LU6
133	Pantothenate Kinase 3	3MK6
134	Suppressor Of Kinetochore Protein 1	3MKS
135	Prephenate Dehydratase	3MWB
136	Farnesyl Pyrophosphate Synthase	3N1V
137	Orf 17	3NJQ
138	Mitogen-Activated Protein Kinase 8	3O2M
139	Camp-Dependent Protein Kinase Regulatory Subunit	3OF1
140	3-Phosphoinositide-Dependent Protein Kinase 1	3OTU
141	Myosin Heavy Chain Kinase A	3PDT
142	Toxin B	3PEE
143	Phospho-2-Dehydro-3-Deoxyheptonate Aldolase	3PG9
144	Udp-Glucose 6-Dehydrogenase	3PJG
145	Camp-Dependent Protein Kinase Type I-Alpha Regulatorysubunit	3PNA
146	Udp-Glucose 6-Dehydrogenase	3PTZ
147	Adenosine Receptor A2A,Lysozyme Chimera	3QAK
148	Nmda Glutamate Receptor Subunit	3QEM
149	Ribonucleotide Reductase R1 Protein	3R1R
150	Ribonucleoside-Diphosphate Reductase Large Chain 1	3RSR
151	Dihydrodipicolinate Synthase	3TCE
152	Probable Dna Double-Strand Break Repair Rad50 Atpase	3THO
153	Insulin-Degrading Enzyme	3TUV
154	6-Phosphofructokinase Isozyme 2	3UMO
155	Glutaminase Kidney Isoform, Mitochondrial	3UO9
156	Glutaminase Kidney Isoform, Mitochondrial	3VOZ
157	Penicillin Binding Protein 2 Prime	3ZG0
158	Nickel And Cobalt Resistance Protein Cnrr	3ZG1
159	Glucose-1-Phosphate Thymidylyltransferase	3ZLK
160	Choline Kinase Alpha	3ZM9
161	Uridylate Kinase	4A7W
162	Lysine Acetyltransferase	4AVC
163	Non-Structural Protein 4A, Serine Protease Ns3	4B6E
164	3-Oxoacyl-[Acyl-Carrier-Protein] Reductase Fabg	4BNU
165	Udp-N-Acetylglucosamine Pyrophosphorylase	4BQH
166	Nad-Dependent Protein Deacetylase Sirtuin-3,Mitochondrial	4C7B

Continued on next page

Table S1 – continued from previous page

No.	Protein Name	PDB ID
167	Integrase	4CIG
168	Adenylate Cyclase Type 10	4CLL
169	Glucokinase	4DCH
170	Camp-Dependent Protein Kinase Catalytic Subunit Alpha	4DFZ
171	Rac-Alpha Serine/Threonine-Protein Kinase	4EJN
172	Peld	4EU0
173	Neurolysin, Mitochondrial	4FXY
174	Aspartate Carbamoyltransferase Catalytic Chain	4FYV
175	Plasminogen Activator Inhibitor 1	4G8O
176	Dihydrodipicolinate Synthase	4HNN
177	Prmt3 Protein	4HSG
178	Pyruvate Kinase 1	4HYW
179	Magnesium Transport Protein Cora	4I0U
180	Mucosa-Associated Lymphoid Tissue Lymphoma Translocationprotein 1	4I1R
181	Heat Shock 70Kda Protein 1A Variant	4IO8
182	Isocitrate Dehydrogenase [Nadp], Mitochondrial	4JA8
183	Citrate Synthase	4JAF
184	Chaperone Protein Dnak	4JN4
185	N-Acetylglutamate Kinase / N-Acetylglutamate Synthase	4KZT
186	Gtpase Kras	4LUC
187	Copper-Sensitive Operon Repressor (Csor)	4M1P
188	Chimera Protein Of C-C Chemokine Receptor Type 5 Andrubredoxin	4MBS
189	Ubiquitin-Conjugating Enzyme E2 R1	4MDK
190	Dna Double-Strand Break Repair Rad50 Atpase	4NCJ
191	Udp-N-Acetylglucosamine 2-Epimerase	4NES
192	Dihydropteroate Synthase	4NIR
193	Cytosolic Imp-Gmp Specific 5'-Nucleotidase	4OHF
194	Metabotropic Glutamate Receptor 5, Lysozyme, Metabotropicglutamate Receptor 5 Chimera	4OO9
195	Bira Bifunctional Protein	4OP0
196	Soluble Cytochrome B562, Metabotropic Glutamate Receptor 1	4OR2
197	Deoxycytidylate Deaminase	4P9D
198	Phosphofructokinase	4PFK
199	Vitamin D3 Receptor A	4Q0A
200	Isoprenyl Transferase	4Q9O
201	Deoxynucleoside Triphosphate Triphosphohydrolase Samhd1	4QFX
202	E3 Ubiquitin-Protein Ligase Rnf146	4QPL
203	Pyruvate Carboxylase	4QSK
204	Prostaglandin G/H Synthase 2	4RUT
205	Eukaryotic Translation Initiation Factor 4E	4TPW
206	Cystathionine Beta-Synthase	4UUU
207	Chorismate Mutase	5CSM

S2. Data and Results' Analysis

S2.1. t-SNE Distribution of Pocket Features

The features of the t-SNE pocket were further analyzed to see their classes, weights, and distance distributions. Allosteric pockets are mainly close to the modulator and have an average size of ∼110 kDa. Refer to the figures S1, S2, and S3.

S2.2. Model Analysis on Confusion Matrix - FP vs. FN

False negatives share some similarities with true positives i.e., large volumes (3754.79 ± 3394.02 Å^3) and high residue counts (16.43 ± 9.52), which indicates that the model struggles with complex, larger binding sites. False positives show intermediate characteristics, with volumes (2878.87 ± 2990.05 Å^3) and residue counts (13.80 ± 10.38) falling between typical binding and non-binding pockets. This pattern is supported by substantial effect sizes between false positives and true negatives for both volume (Cohen's $d = 0.77$) and number of residues (Cohen's $d = 1.14$). Hydrophobicity shows minimal variation across all categories (p=0.8891 in Kruskal-Wallis test), showing that it is not a decisive factor in the model's predictions. The model appears to struggle most with "borderline" cases where size and volume metrics fall between typical binding and non-binding ranges, and with complex pockets having higher residue counts. While the small number of false predictions indicates good overall performance, these edge cases where multiple features do not clearly align with typical characteristics represent the model's main weakness. The correlation

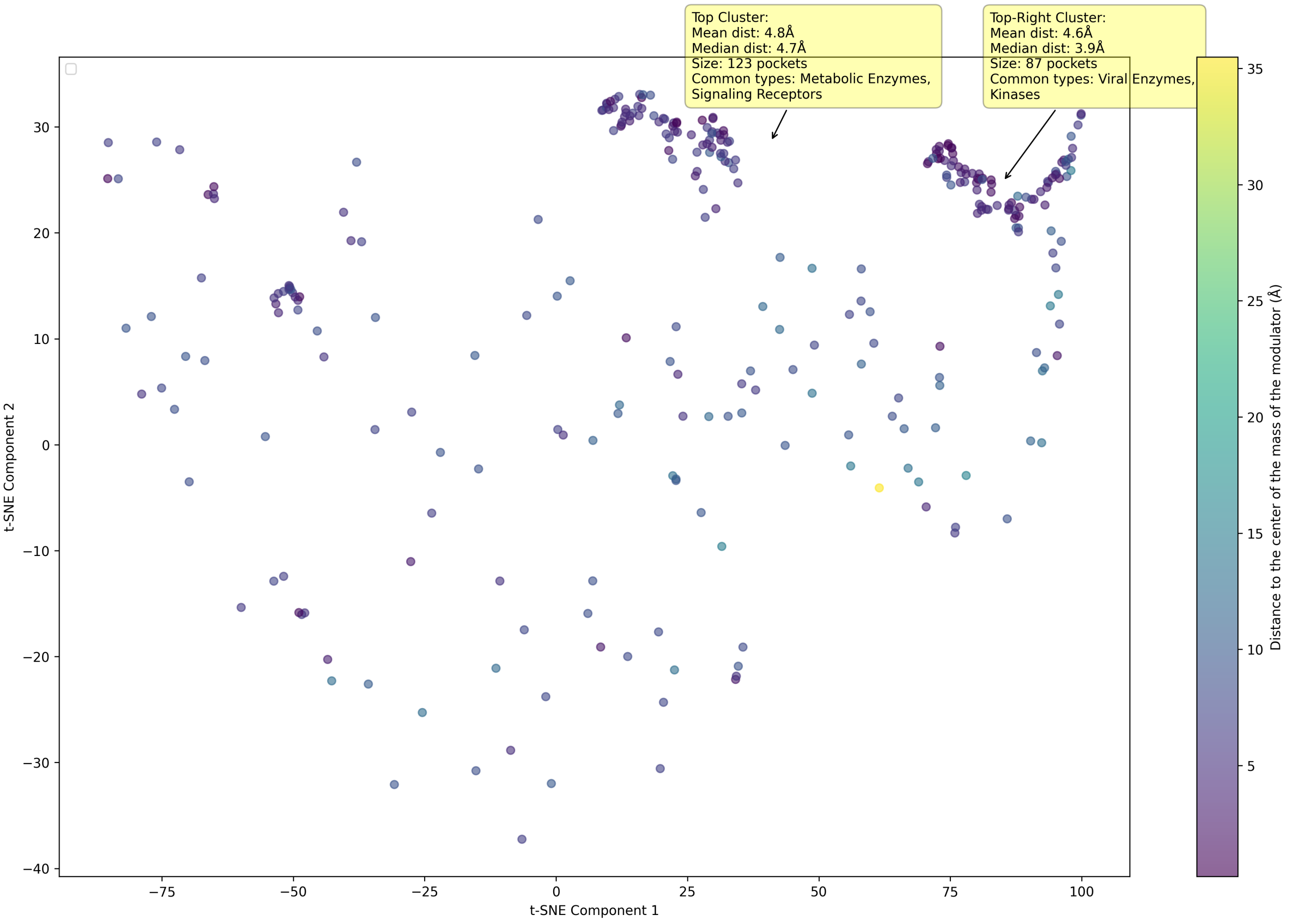

Fig. S1. Top and Top-right Clusters of Allosteric Pockets - Axes' labels are random names that represent the two features given by t-SNE analysis.

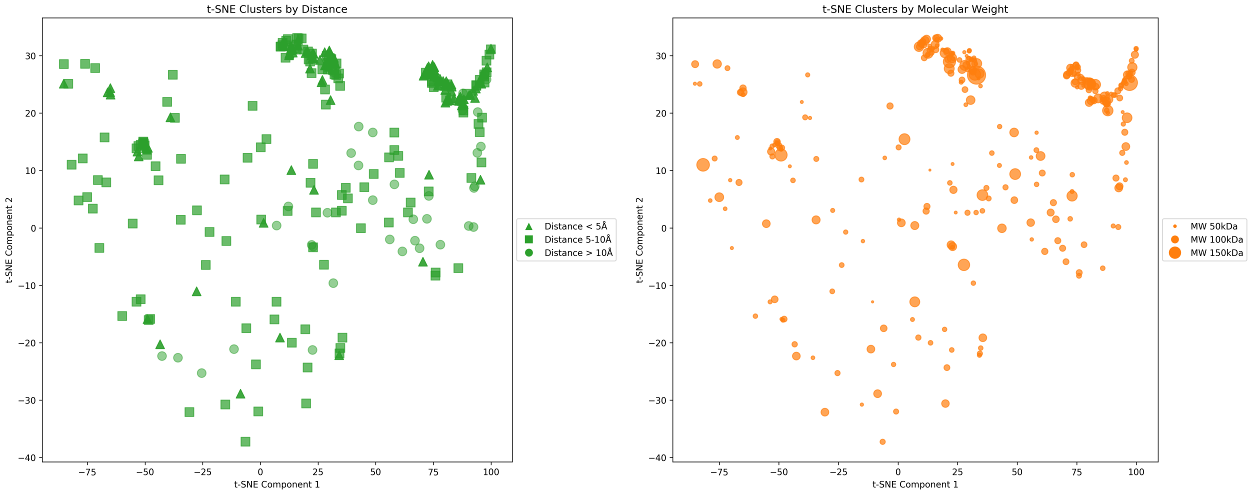

Fig. S2. Allosteric Pocket Clusters - Average mean weight (MW) for allosteric pockets is 110 kDa, however, the top-right cluster has a lower median distance (3.87 Å) between the center of mass of a pocket and the center of mass of its modulator compared to the top cluster (4.65 Å). (Axes' labels are random names that represent the two features given by t-SNE analysis.)

matrix shows hydrophobicity is not highly correlated with other metrics (*size*, *volume*, and *number of residues*). These trends are given in figures S4, and S5.

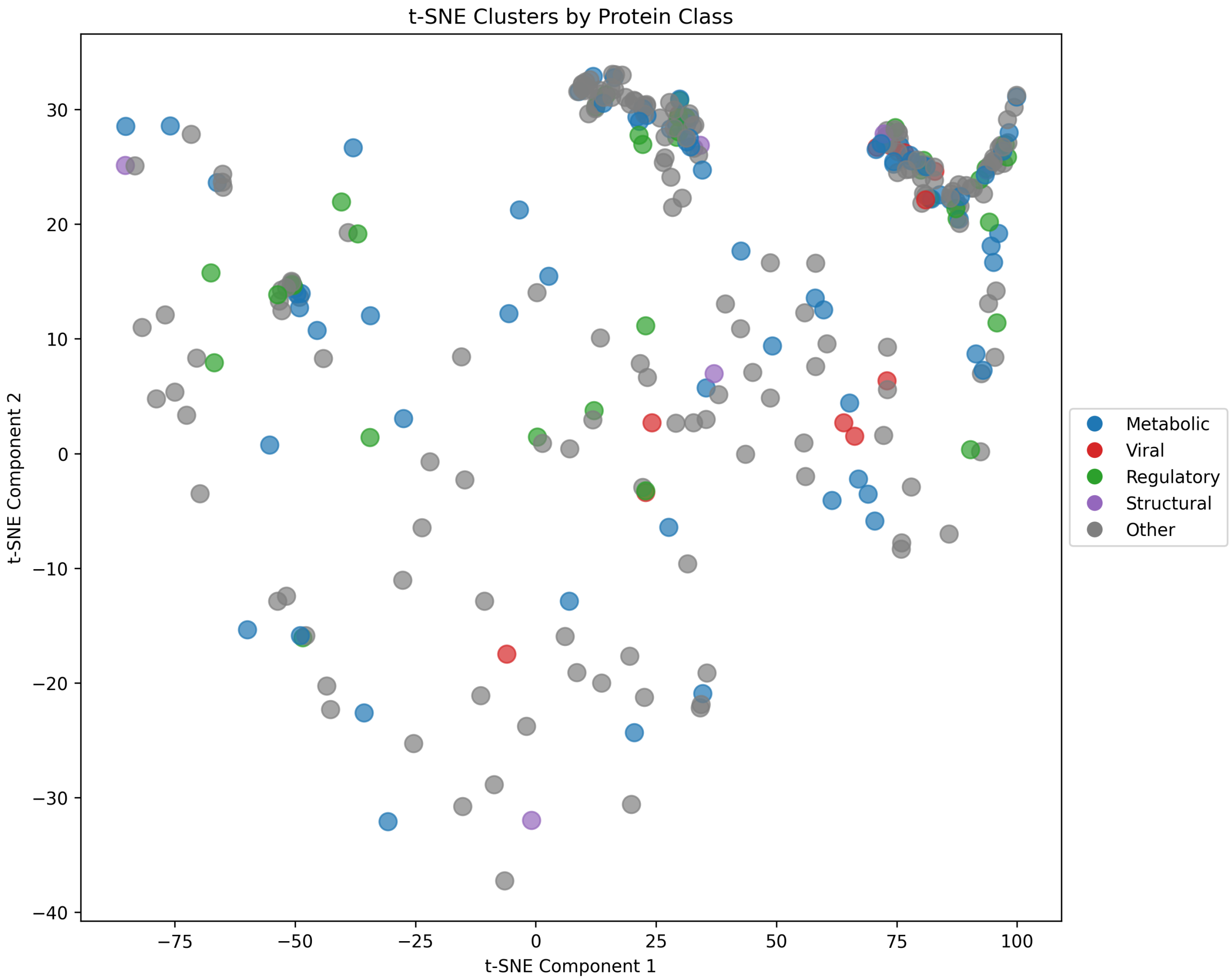

Fig. S3. Allosteric Pocket Clusters with Protein Classifications - Top right cluster's modulator types mostly consist of ATP, nucleotide analogs (e.g., cAMP), and small-molecule inhibitors (e.g., BPTES), however in the case of top-right one, it is mostly viral inhibitors (e.g., HIV RT inhibitor 7), antibiotics (e.g, ceftaroline), and kinase modulators. (Axes' labels are random names that represent the two features given by t-SNE analysis.)

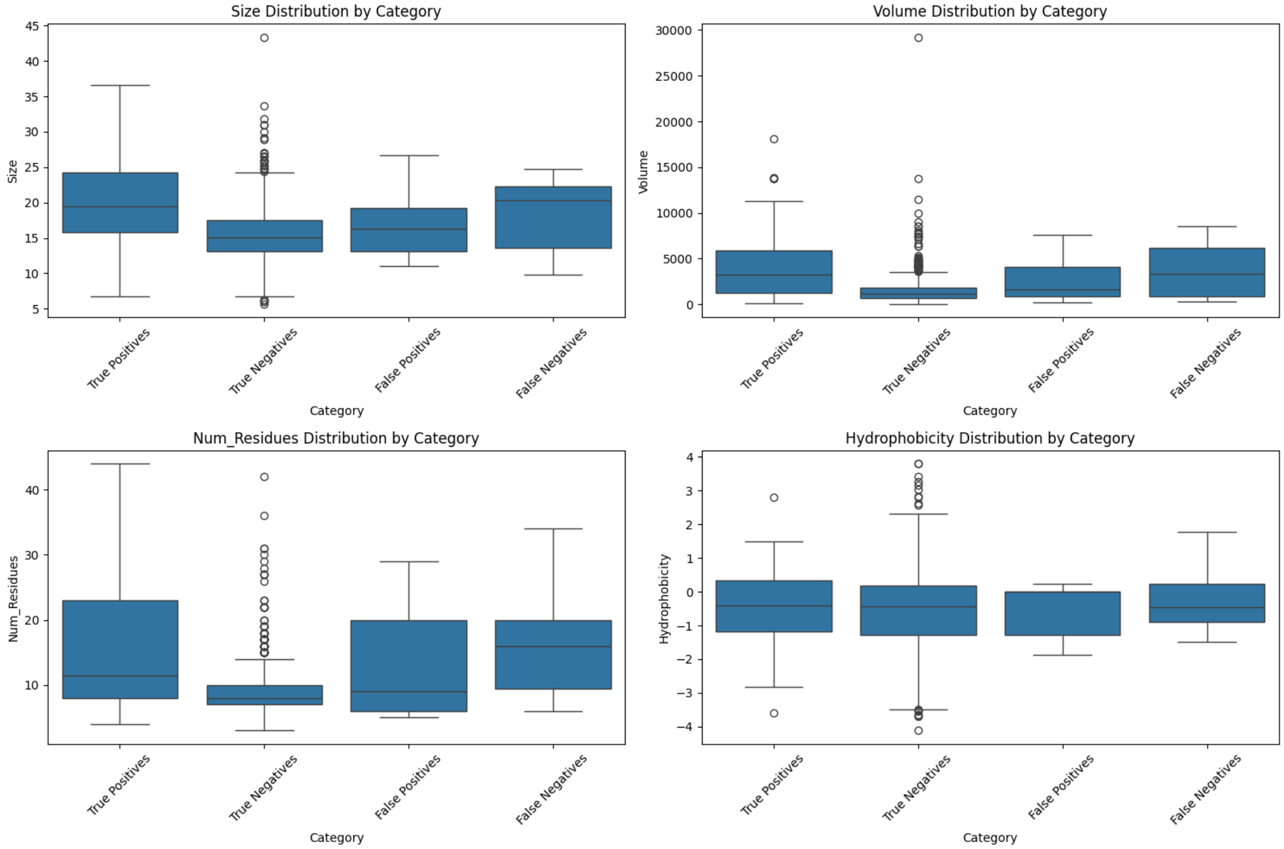

Fig. S4. Pockets Distribution by Size, Volume, Hydrophobicity, and Number of Residues - Size represents the maximum pairwise distance between any two points in the pocket (in Å). Volume is calculated as the product of the bounding box dimensions containing all pocket points (in $Å^3$). Num_Residues indicates the count of amino acid residues lining the pocket.

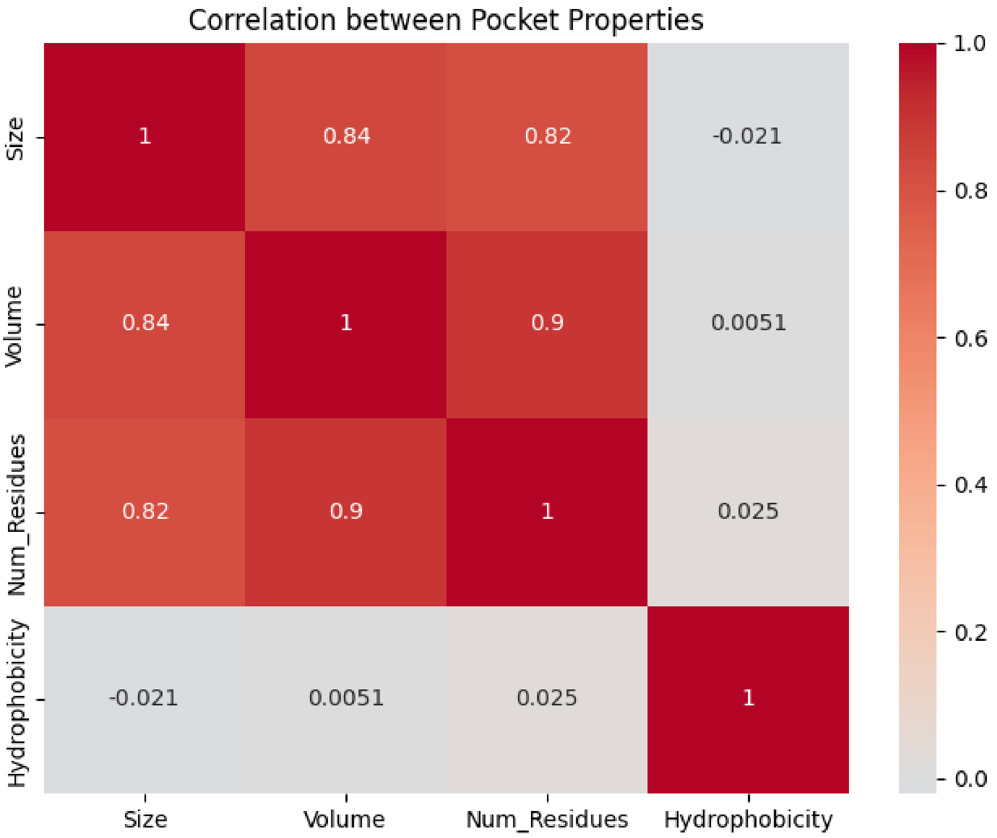

Fig. S5. Correlation heatmap showing the relationships between different pocket properties. Strong positive correlations are observed between Size, Volume, and Number of Residues (0.82-0.9), indicating these geometric properties are highly interrelated. In contrast, Hydrophobicity shows negligible correlations with all other properties (correlation coefficients between -0.021 and 0.025), suggesting that the chemical nature of the pocket is independent of its physical dimensions.
